# Lateral clavicle fractures: grid pattern arrangement of screws in the lateral fracture fragment reduces the cut-out

**DOI:** 10.1007/s00402-025-06027-z

**Published:** 2025-08-12

**Authors:** Stefanie Hoelscher-Doht, Sophia Scheible, Maximilian Heilig, Eva Kupczyk, Rainer H. Meffert

**Affiliations:** 1 Department of Trauma, Hand, Plastic and Reconstructive Surgery, University Hospital Würzburg, Wurzburg, Germany; 2 Department of Trauma and Orthopaedic Surgery, BG Unfallklinik Frankfurt Am Main, Frankfurt am Main, Germany

## Abstract

**Introduction:**

Lateral clavicle fractures often need to be stabilized by a plate osteosynthesis and due to the acting high forces a cut-out of the screws of the lateral fracture fragment can occur. New plates enable to place anterior screws in addition to the screws placed from the top of the clavicle. This experimental in-vitro study will determine whether they have a substantial biomechanical effect.

**Materials and methods:**

In synthetic bones, lateral clavicle fractures were created and stabilized in 4 different groups: In Group A, a lateral clavicle plate was fixed with 3 screws in the lateral fracture fragment. In group B, the same type of plate was fixed with additionally two screws from anterior in the lateral fragment. In group C, a coraco-clavicular banding was added to the fixation method of group B. A similar plate from another company was used in group D with a screw fixation method comparable to group A. In a material testing machine, the specimens were loaded by dynamic and static tests. The mode of failure and pull-out forces were analyzed.

**Results:**

In the dynamic testing phase, five specimens failed already in group A and B, whereas in group C 11 specimens survived the cyclic tests. Lateral fractures and screw cut-out appeared in the static tests mostly in group A and B. In contrast, in group C, ten of eleven specimens failed by a medial fracture at the plate end. No significant differences were determined in-between groups for the displacement recorded by the optical system, even, when group A revealed the highest values of the groups A-C. In group D, the specimens showed an early screw cut-out of the lateral fracture fragment, and all failed during the dynamic testing phase.

**Conclusions:**

The use of additional screws from anterior led in a significant lower cut-out and higher biomechanical stability at the lateral clavicle regarding axial tensile forces. From a biomechanical point of view, plates for stabilization of lateral clavicle fractures with additional screw holes from anterior and restoring the cc-bands is favorable to standard plates with screws from the top of the clavicle only.

## Introduction

Clavicle fractures are a common injury of the upper extremity with an incidence of from 3 to 10% of all fractures of the shoulder girdle [1, 2]. Most of the fractures occur in the middle third (75%). In 20%, the lateral end is involved and only 5% of all clavicle fractures affect the medial end [1]. Lateral clavicle fractures often are accompanied with a collateral ligament rupture of the coraco-clavicle (cc) ligaments remaining in a high instability. In general, operative and non-operative treatment options must be discussed. The trend towards more frequent surgical treatment in the recent past, particularly for multi-fragmentary fractures and shortening over 2 cm, is due to a frequent pseudarthrosis rate in up to a third of cases in conservative treatment [3, 4].

**Fig. 1:**
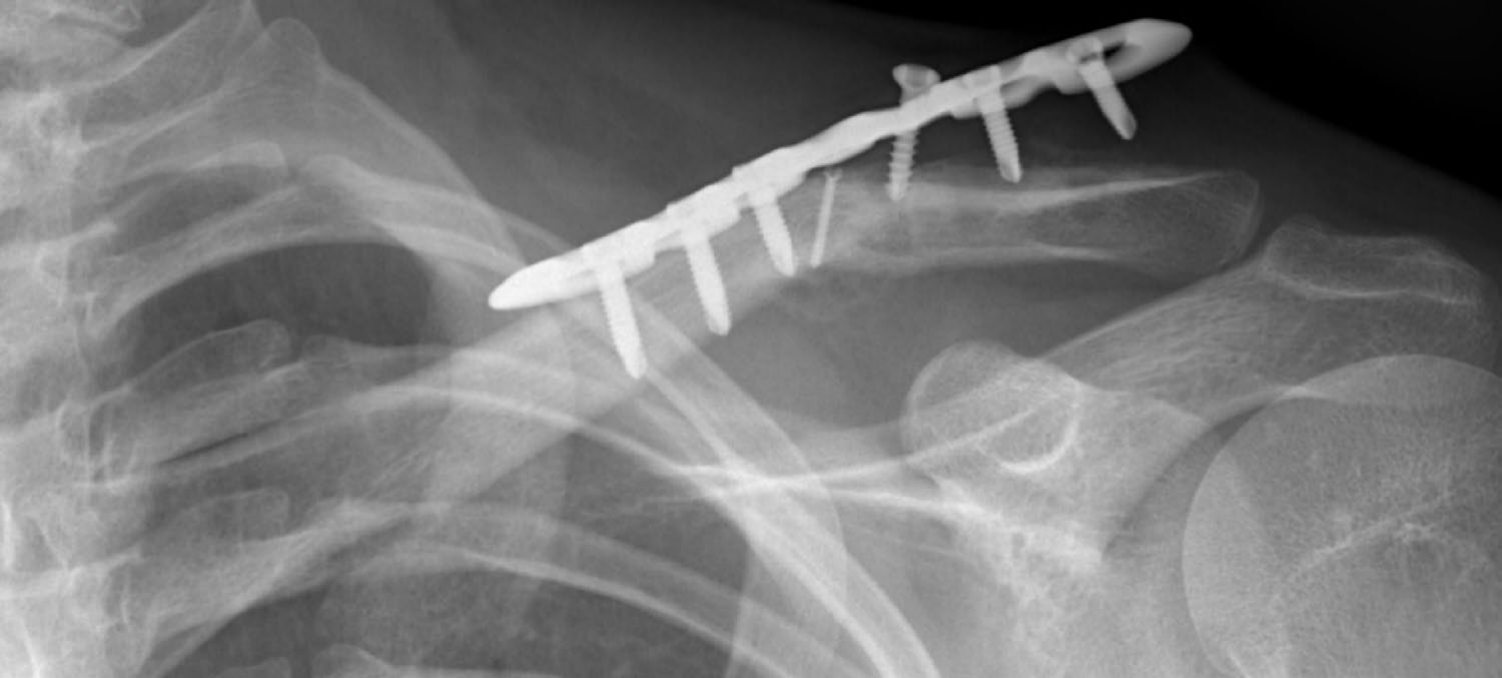
A cut-out of the screws in the lateral fracture fragment like shown in the x-ray can occur in respect to the demanding high forces acting on the lateral end of the clavicle

Therefore, highly instable fractures of the lateral end of the clavicle are often treated surgically: The in the past often used hoo plate had limitations concerning pain, removal of the implant, acromial erosion and rotator cuff tendinopathy [5–7]. An attractive alternative is the stabilisation by a locking plate osteosynthesis with or without an additional banding to address the ligament instability of the cc-joint [8]. Unfortunately, this treatment technique can lead to a pull-out of the screws from the lateral fracture fragment [9–11] (Fig. 1).

New superior lateral clavicle plates are available with a possible banding right through the plate by a plate slot. Additionally, it is possible to place two screw fixations from anterior to posterior so that the screws stabilize the lateral fracture fragment in a 90° angle. From these new features, a higher biomechanical stability is expected. Experimental studies to analyse the primary stability of osteosyntheses in clavicle fractures frequently address fractures of the middle third [12–15]. Biomechanical investigations of fracture fixation of lateral clavicular fractures are less common. *Suter et al.*. provided with a double plating technique in lateral clavicle fractures an interesting alternative with a comparable biomechanical stability to single plate fixation [16]. Yoon et al.. demonstrated an equal stability for 3.5 mm and 2.7 mm screws for locking plates in a biomechanical study [17]. Moreover, a higher stability could be shown for the combination of plate and coracoclavicular tightrope [18, 19] or coracoid button augmentation [20, 21] in highly unstable lateral clavicle fractures in an experimental study. The new plate presented here combines all advantages in one implant: the possibility of inserting additional screws from anterior to posterior and CC banding via a button in the plate. The purpose of this study was to analyse the effectiveness of the biomechanical effect of the placement of the additional screws and the banding.

## Materials and methods

### Specimens and fracture simulation

In sawbones (3408-1 4th generation, Sawbones Europa, Malmö, Sweden) composites bones for biomechanical tests [13, 14, 22–25], lateral clavicle fractures (type Neer 2B) were simulated by a saw. Exact fracture size and edges were ensured by a custom-made 3D-printed saw template to enable a high standardization. A fracture gap of 3.5 mm was created with the intention of simulating an instable fracture type.

### Testing groups

**Fig. 2:**
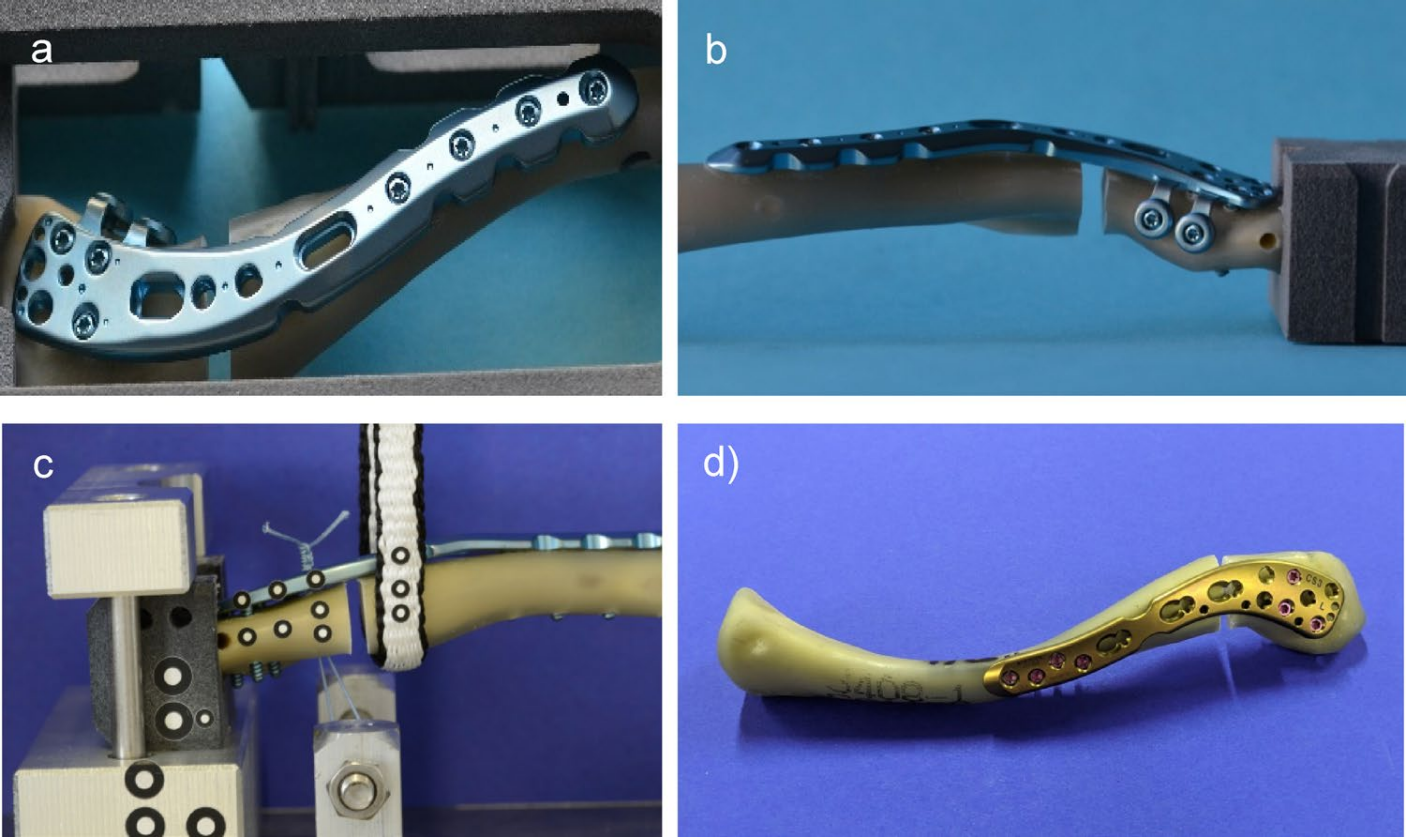
The different osteosyntheses of the 4 groups are shown: In group 1 (**a**) and 4 (**d**), the lateral fragment was fixed with 3 screws from superior only (group 1: Medartis plate; group 4: Synthes plate). In group 2 (**b**) and 3 (**c**), additionally 2 screws from anterior respectively a coraco-clavicular banding stabilized the lateral fracture fragment)

The fractures in 48 synthetic bones were fixed in 4 different stabilization techniques with *n* = 12 per group (Fig. 2): In the first group A, an osteosynthesis by a new lateral clavicle plate (Superior lateral clavicle plate, A-4851.01-04, Medartis AG, Basel, Switzerland) with 3 screws in the lateral fracture fragment was performed. In the second group B, additionally 2 screws from anterior to posterior were placed in the raft technique. In group C, the same fixation of the lateral fracture fragment like in group 2 was performed adding a coraco-clavicular banding by a 2.0 Fiber wire (Arthrex, Naples, USA) through a preformed hole in the plate with a clip. An alternative lateral clavicle plate (Superior lateral clavicle plate (0X.112.013), DePuy Synthes, Johnson&Johnson Medical GmbH, Norderstedt, Germany) fixed with 3 screws in the lateral fracture fragment comparable to group A was chosen in group D. (Table 1). Exact plate positioning and screw insertion were ensured by 3D-printed templates. Due to the long-lasting pre-testing series, an overdrilling of the normally used drill for the screws had to be performed to reproduce a senseful mode of failure like screw pull-out of the lateral fracture fragment. A drill with a diameter of 2.7 mm was used for 2.8 mm screws in group A-C. In group D, the screw holes were drilled 2.6 mm for the 2.7 mm screws.

**Table 1:** The different fixation methods in group 1–4 are shown

	Lateral Clavicle Plate type	Screws from superior	Screws form anterior	CC-Banding
Group A	Medartis Superior lateral clavicle Plate	3	-	-
Group B	Medartis Superior lateral clavicle Plate	3	2	-
Group C	Medartis Superior lateral clavicle Plate	3	2	+
Group D	Synthes superior lateral clavicle 5-hole plate	3	-	-

### Biomechanical test set-up and parameters of interest

**Fig. 3:**
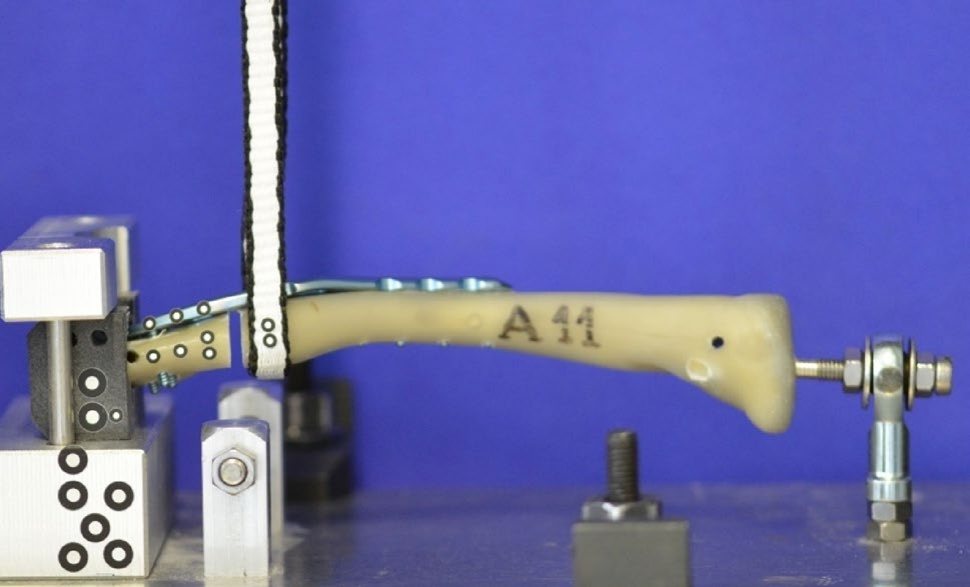
In the biomechanical test set-up, tensile forces on the lateral fracture fragment were simulated

**Fig. 4:**
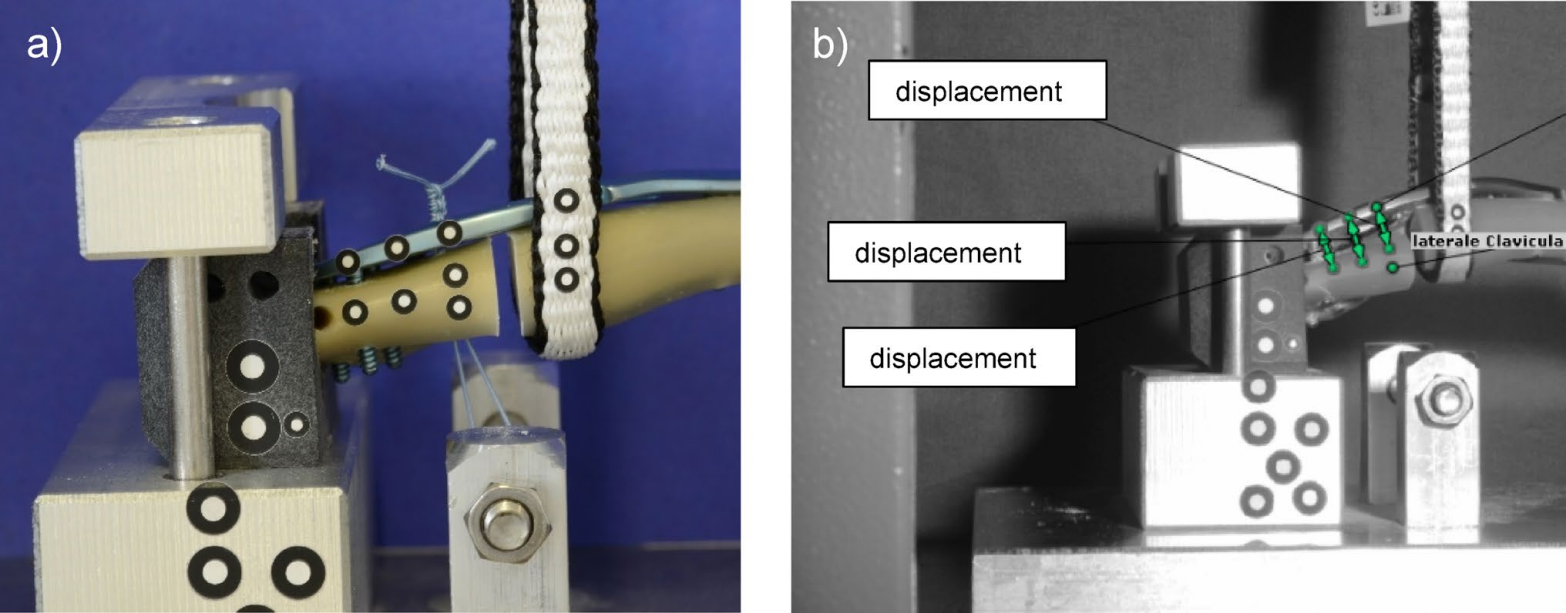
Optical analysis was performed by an optical measuring system (Aramis, Carl Zeiss GOM Metrology GmbH, Jena, Germany): **a** standardized pattern of markers at the lateral end of the clavicle. **b** three relative movements between the plate and bone were measured by the markers (relative movement 1: lateral end; relative movement 1: directly lateral of the fracture gap; relative movement 2: between movement 1 and 3

In a very elaborate pre-testing series with different testing modalities like bending and tensile tests, the final test set-up was determined based on the study by *Steffanoni et al.* [26] and considering daily living activities [27]: The specimens were fixed in a custom-made device with limited degrees of freedom on the medial and lateral side (Fig. 3). A tensile load was applied laterally on the clavicle by a sling. A pre-load of 10 N was followed by a dynamic testing series with 3000 cycles from 100 N to 450 N with a crosshead speed of 50 mm/min. Static tests were also performed to determine maximum load and stiffness with a switch-off threshold of 80% maximum load. By an optical system (ARAMIS 3D Professional, Carl Zeiss GOM Metrology GmbH), the cut-out of the lateral screws was analyzed during dynamic testing (Fig. 4).

### Optical analysis

For detailed analysis of the movements and loosening of the screws, especially on the lateral fracture fragment, an optical analysis was performed during the biomechanical tests using an optical system (Aramis, Carl Zeiss GOM Metrology GmbH, Jena, Germany). Reference markers distributed in a standardized pattern (Fig. 4a) were used to determine the exact plate displacement during the different loading conditions. For this purpose, the optical measuring system and universal testing machine were connected to each other via an analog channel. After loading, the markers were used to calculate the distances between bone and plate (green arrows, Fig. 4b) at three points on the lateral fracture fragment. A reference image was recorded before the start of the dynamic test phase and the relative distance in relation to the reference image was measured with the software (ARAMIS Professional 2019, Carl Zeiss GOM Metrology GmbH, Jena, Germany).

### Parameters of interest

Parameters of interest were the three relative movements (displacements) of the lateral fracture part in relation to the plate measured by the optical system during the dynamic testing phase. The total displacement in the dynamic tests was also recorded by the traverse and the software of the material testing machine (testxpert III, Zwick Roell, GmbH, Ulm, Germany).

Maximum load and stiffness were determined in the static testing phase. Modes of failure during dynamic and static testing phases were analyzed.

### Statistics

Statistical analysis was performed with SPSS (SPSS 28.0, IBM^®^, Statistics Program) in cooperation with statistical consultation service for research projects of the Institute for Epidemiology and Biometry (Julius-Maximilians-University of Würzburg, Germany). All parameters of interest were tested for normal distribution with a Shapiro-Wilk-Test and a Kolmogorov-Smirnov-Test and for homogeneity of variance with a Levene-Test. ANOVA or ANOVA-Welch tests followed depending on the outcome of the Levene-Test. As post-hoc-tests, Bonferroni and Games-Howell were used also depending on the homogeneity of variances for the analyzed parameter.

## Results

### Mode of failure in the dynamic testing phase

The biomechanical tests comprised a dynamic testing phase with 3000 cycles and a static testing phase with load-to-failure tests after finishing the dynamic phase. During the cyclic loading, failure with fracture of the bone or screw cut out of the lateral fracture fragment occurred in some cases (Table 2). In detail, in group A and B, five specimens failed during the dynamic testing in each of the groups. In contrast, in group C, only one specimen did not complete the cyclic loading. Group D demonstrated very early failure during dynamic testing with six of the twelve specimens not even completing one cycle. Eleven of the specimens revealed a complete screw cut-out out of the lateral fracture fragment. In one case, a lateral fracture occurred.

**Table 2:** Number and type of failure of specimens during the dynamic testing phase of all groups

Group	Number of failed specimens	Lateral fracture	Medial fracture	Lateral and medial fracture	Lateral screw cut-out
A	5	2	1	1	1
B	5	3	2	-	-
C	1	1	-	-	-
D	12	1	-	-	11

### Mode of failure in the static testing phase

**Fig. 5:**
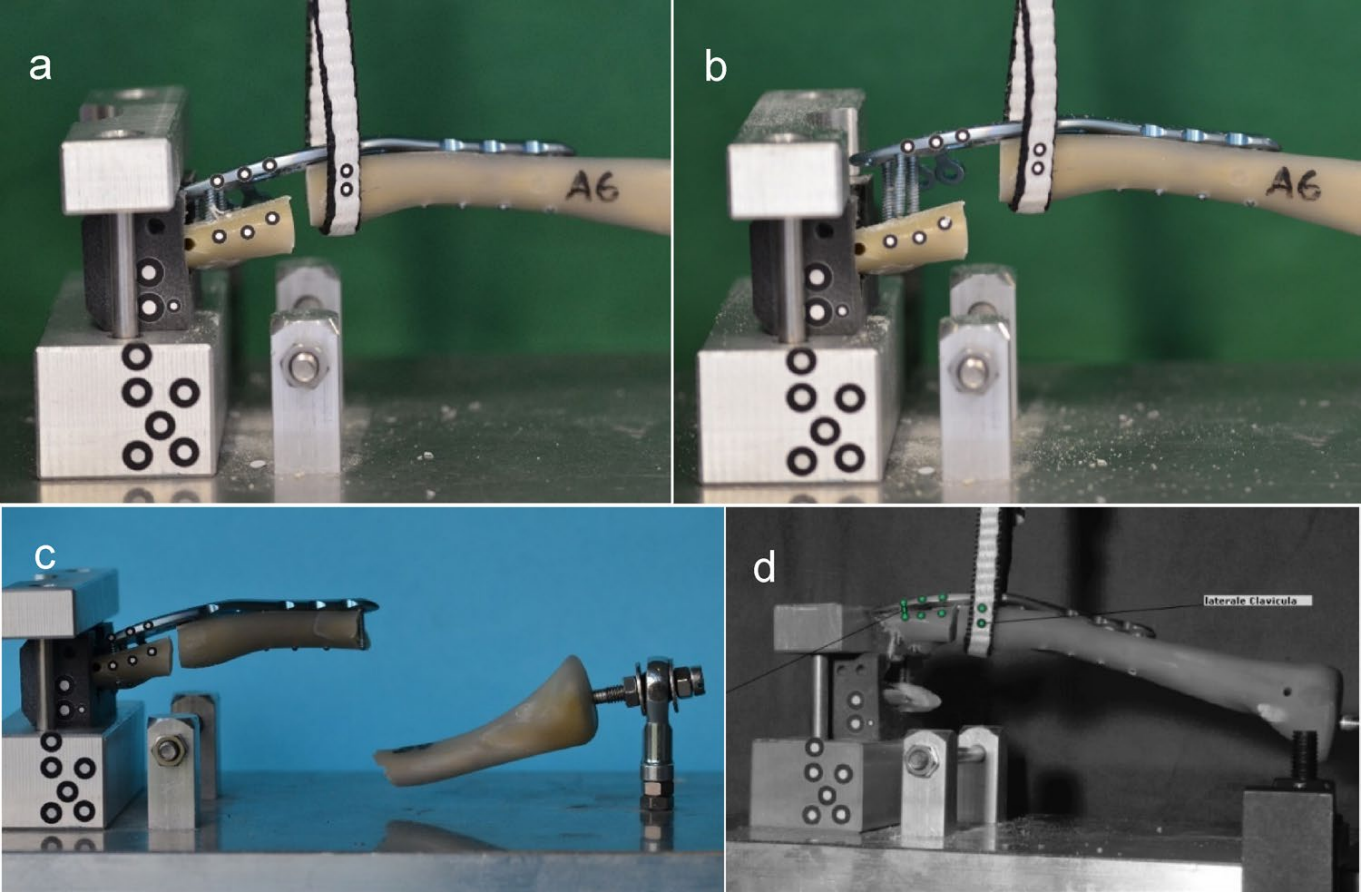
Different failure modes: **a** lateral screw loosening after the dynamic test phase and **b** lateral screw pullout after the load-to-failure tests. **c** failure mode of medial fracture at the plate end and **d** failure mode of lateral fracture around the screws

The seven remaining specimens of group A revealed a screw-loosening during the dynamic testing phase (Fig. 5a), and in the subsequent static tests, four showed a failure mode of complete screw-pull-out (Fig. 5b) and three broke medially (Fig. 5c). In group B, seven specimens underwent the static testing phase, and all demonstrated a medial fracture of the bone at the plate end (Fig. 5c). In group C, in one case, a lateral fracture around the screw occurred (Fig. 5d). The remaining 10 specimens of this group revealed a medial fracture at the plate end. In group D, none of the specimens completed the cyclic testing phase and so none reached the load-to-failure tests. Number and type of failure after the static tests are demonstrated in Table 3.

**Table 3:** Number and type of failure of specimens during the static testing phase of all groups

Group	Lateral fracture	Medial fracture	Lateral and medial fracture	Lateral screw cut out
A	-	3	-	4
B	-	7	-	-
C	1	10	-	-

### Dynamic testing phase

#### Analysis of the optical system

**Fig. 6:**
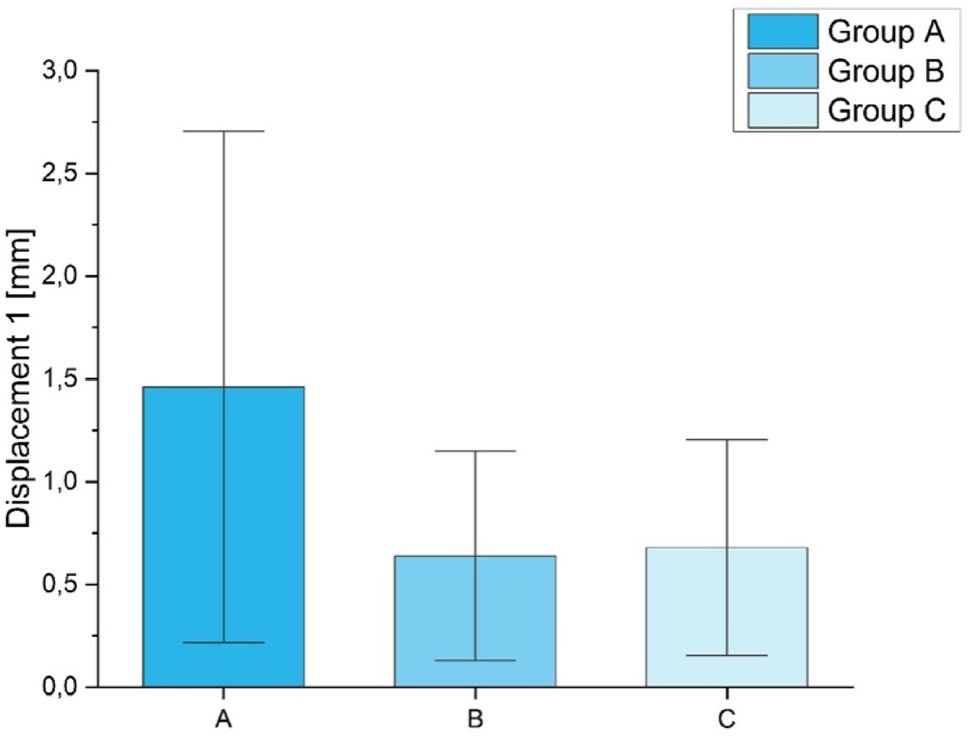
Displacement 1 with standard deviation. Group A with the highest mean, however the difference to the other groups was not significant (*p* = 0.37)

Despite some test specimens failed before completing the cycles, the screw loosening out of the lateral fracture fragment of the groups A, B and C was evaluated. Therefore, the three distances (displacements) between the plate and the lateral fracture fragment were analyzed like described in the section *Materials and Methods*,* Optical analysis (*Fig. 4b*)*. No significant differences (*p* = 0.37) could be detected between the experimental groups A-C. For *displacement 1* (Fig. 6) the mean values were 1.46 ± 1.24 mm for group A, 0.64 ± 0.51 mm for group B and 0.68 ± 0.52 mm for group C. For *displacement 2* the mean values were 1.69 ± 1.40 mm for group A, 0.88 ± 0.66 mm for group B and 0.95 ± 0.68 mm for group C. For *displacement 3*, the following values were determined: 1.93 ± 1.50 mm for group A, 1.02 ± 0.72 mm for group B and 1.13 ± 0.75 mm for group C. No differences were found in-between groups (*p* = 0.40).

#### Analysis of displacement recorded by the material testing machine program

In addition to the displacement analysis with the optical system with the Aramis software, the displacement *testexpert* of the complete construct of the specimens was determined by the testexpert software of the material testing machine (testexpert III, Zwick Roell GmbH, Ulm, Germany).

**Fig. 7:**
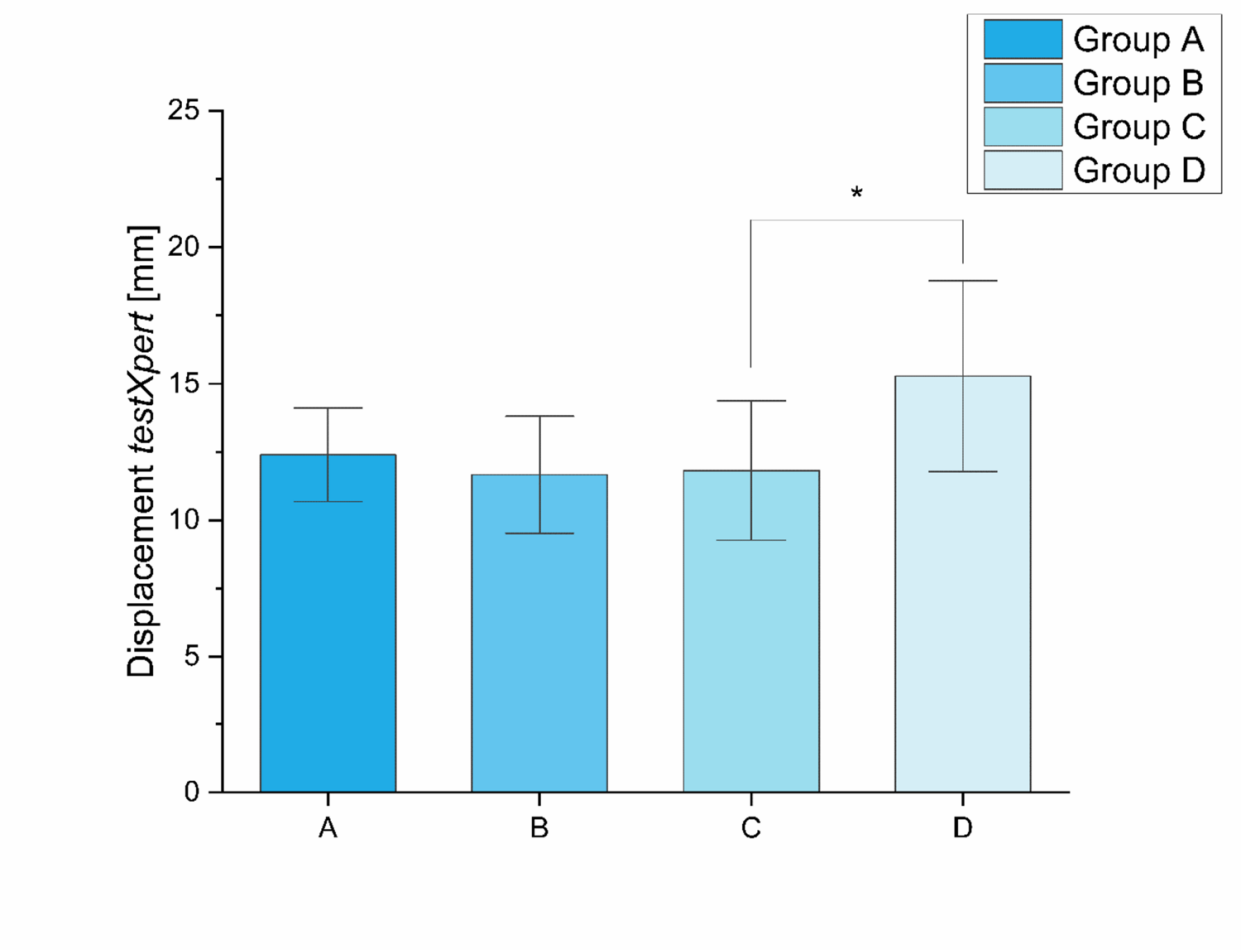
Displacement *testexpert* recorded by the material testing machine: Group D showed a significantly higher maximum displacement than group C (*p* = 0.02) as marked (*)

The mean values for group A were 12.40 ± 1.71 mm, for group B 11.66 ± 2.15 mm, for group C 11.82 ± 2.56 mm, and for group D 15.28 ± 3.50 mm (Fig. 7). A significant difference was detected between groups C and D (*p* = 0.02).

### Static testing phase

**Fig. 8:**
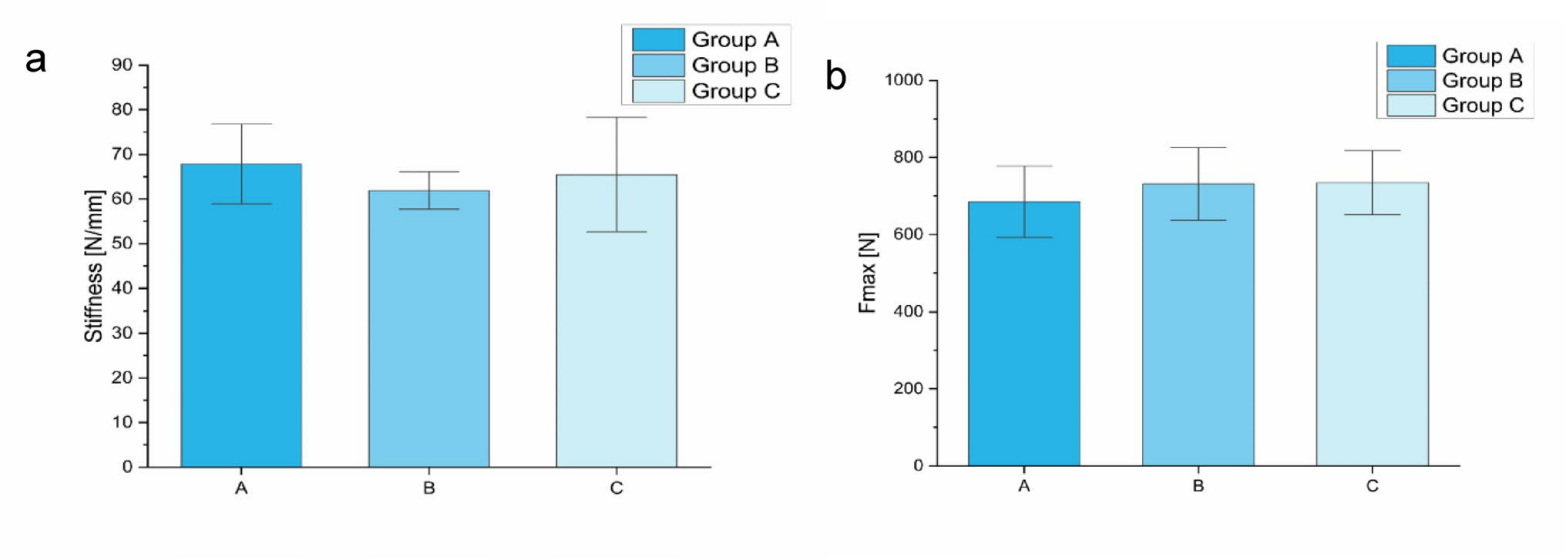
The results of the static tests are demonstrated: (**a**) For the stiffness, no significant differences were detected in-between the groups (*p* = 0.34). **b** For the maximum force, no significant differences could be shown (*p* = 0.54)

In the load-to-failure tests, maximum load and stiffness were determined for group A-C. The mean values for the stiffness were 67.9 ± 9.0 N/mm for group A, 62.0 ± 4.2 N/mm for group B, and 65.5 ± 12.8 N/mm for group C (Fig. 8a). For the maximum load, group A revealed a mean force of 685 ± 93 N, group B a mean force of 731 ± 95 N, and group C 735 ± 84 N (Fig. 8b). No significant differences were detected in-between groups (stiffness: *p* = 0.34; maximum load: *p* = 0.54).

Figure 8: The results of the static tests are demonstrated: (a) For the stiffness, no significant differences were detected in-between the groups (*p* = 0.34). (b) For the maximum force, no significant differences could be shown (*p* = 0.54).

## Discussion

High forces are already acting on the clavicle during daily routine [27]. Osteosynthesis in the context of fracture treatment is therefore faced with high demands so that no implant failure occurs. New implants represent an interesting alternative to previous ones. Using additional plate holes, in the grid technique, superior screws with anterior screws can ensure even higher stability of the fixation of the lateral fracture fragment. In the present study, the objective was to determine the biomechanical advantage of the additional screws and cc-banding with fixation in the plate.

In order to test the primary stability of osteosyntheses on the lateral clavicle, a suitable test setup first had to be established. Based on the literature on biomechanical studies at the lateral end and on computer-based models such as finite element models, a detailed series of pre-tests was carried out to investigate the validity of different test modalities [17–21, 28–32]. Anterior and posterior translation tend to be negligible in the model, leading to a focus on compression [26] and tensile forces [17, 21, 28, 29, 32]. The tensile forces, in particular, predominate at the lateral clavicle, which is why the clinically observed screw pull-outs from the lateral fracture fragment can occur (Fig. 1). In the end, a model with tensile forces was chosen in which the clinical failure mechanism could be demonstrated. However, this could only be achieved by drilling larger holes closer to the screw diameter than usual in clinical practice. In our opinion, models in which only a fracture of the medial end, despite differences in fixation, occurs (like in divers test set-ups of our pre-testing series) do not provide any biomechanical insights into the differences between osteosyntheses that can be transferred to the clinic. Both, computer simulations [26] and pre-tests with increasing or continuous force levels, were also used to determine the loading level for dynamic testing. The maximum load of 450 N, selected for the final tests, corresponds to forces that occur at the transition area from the middle to the lateral third of the clavicle, when lifting a 1 kg weight (anteversion) up to the horizontal [26].

For the first time, new superior lateral clavicle plates are available with a possible banding right through the plate by a plate slot. Furthermore, it is possible to place two screw fixations from anterior to posterior additionally to the screws from superior, so that the screws stabilize the lateral fracture fragment in a 90° angle grid pattern. To analyse the biomechanical advantages of this new features of the lateral clavicle plates, was the aim of this presented research project. We expected, especially from the combination of CC-banding through the plate and the additional screws from anterior, a distinctly higher primary stability to avoid screw-loosening or screw cut-out of the lateral fracture fragment like seen in clinical practise.

This biomechanically higher stability was demonstrated in the failure modes in the dynamic and static testing phases: In group A, screw pull-out of the lateral fracture fragment was seen with the highest values of all displacements, even though not significantly different to group B and C. In the load-to-failure tests, in group A in majority of the cases, the mode of failure was a complete cut-out of the lateral screws in group A, whereas in group B and C most of the specimens failed by a medial fracture at the plate end. In group C, only one specimen did not complete the cyclic loading, while in contrast, in group A and B five specimens in each group already failed during the dynamic testing phase. The more the lateral fracture fragment is fixed by additional screws as in group B and by CC-banding in group C, the more likely a failure mode with stress peak and consecutive fracture at the medial plate end is observed. In addition, due to the better fixation of the lateral fragment as in group C, the specimens survived the dynamic testing better than in groups A and B. The comparison under the highly standardized test conditions between groups A, B and C is the most meaningful. In the three mentioned groups, the same plate and screws with the same over-drilling 0.1 mm less than the screw diameter were applied for fracture stabilization. The test model had to be adapted to the testing conditions with difficult simulation of the acting forces at the lateral clavicle end and use of synthetic bones, in which a screw cut-out is meant to be nearly impossible to simulate. We therefore consider ourselves lucky that we were able to establish a standardized test model in a long pre-test phase. By using synthetic bones, and sawing and drilling templates (with a 3D printing accuracy of 0.01 mm), a high standardization of the test model was generated, which is highly superior to the use of human bone to draw conclusions from the biomechanical tests. However, due to the need to over-drill the screw holes to a larger diameter than is normally done during surgery, the test model also has a limitation in that comparison between the two different plate and screw systems (group A and D) is only possible to a very limited extent. In group D, regularly in contrast to group A with a comparable plate and screw fixation, a cut-out of the screws in the lateral fracture fragment was seen, suggesting, that the screw thread, which is a little bit smaller in group D than in group A, is crucial to avoid early screw-loosening. Nevertheless, a direct translation from the comparison of group A and D to clinical practice is not possible reflecting the over-drilling of the screw holes. Anyway, in an elaborate test model, it was possible to gain fundamental initial insights into the biomechanical advantages of additional screwing and addressing of the ligament instability with fixation of the banding by the plate. By extending the fixation options of the lateral fragment, it is better biomechanically stabilized and, as the results demonstrated, the forces generated under tensile loads are more likely to be transmitted to the medial end of the plate. Interestingly, no loosening of the screw head out of the lateral plate was seen in any mode of failure of the groups A-C. Due to the laterally lower plate design with significantly less pressure during locking of the screw heads in contrast to the medial plate end, lateral locking of the screws represents a potential weak point under loading.

### Limitations

The findings of the study relate to healthy human bone, the biomechanical properties of which were optimized using synthetic bones fabricated for biomechanical in-vitro studies [33]. This is well in agreement considering the mainly affected group of 20–40-year-old adults regarding the incidence of lateral clavicle fractures [34, 35]. How osteoporotic bones react under biomechanical testing conditions must been proved in further cadaver studies. Furthermore, the forces acting on the clavicle had to be reduced to a minimum for the model in the laboratory and for the complex test pattern, the screws in the lateral fragment were drilled with a larger drill than is normally used. This limits the comparability of the two plates. Nevertheless, the biomechanical test model can provide us with relevant information about the differences and advantages of using screws in the grid technique and additional banding.

## Conclusions

In conclusion, this study has successfully established a biomechanical test model in which screw loosening and screw cut-out from the lateral fracture fragment could be demonstrated under tensile loading. First information regarding the higher stability and the redistribution of the peak stress towards the medial end of the plate due to the new plates with optimized lateral fixation could be obtained from the study.

## Data Availability

Data is provided within the manuscript.
